# Our Birthday

**Published:** 1896-06

**Authors:** 


Our Birth-day.—This issue completes the eleventh year of unprecedented success of the "Red Back." We take occasion to tender our grateful acknowledgements to our patrons who have so generously supported the efforts to give Texas the *best medical journal* that can be gotten out. With renewed courage the management sets in on the twelfth round, determined to keep it up to the top notch of excellence. Our subscription list has grown so large lately that we find it necessary to cut off quite a number of our exchanges—not needed in the business—and we will begin with next issue a monthly edition increased twenty per cent. Advertisers will please take notice. The "Red Back" has the innings, though it does not claim—as one of our Texas contemporaries did lately that we issue "nearly six thousand."

Accept our best thanks and consider our bow as being here made.

